# Antibiotics Limit Adaptation of Drug-Resistant Staphylococcus aureus to Hypoxia

**DOI:** 10.1128/aac.00926-22

**Published:** 2022-11-21

**Authors:** Rebecca C. Hull, Rosanna C. T. Wright, Jon R. Sayers, Joshua A. F. Sutton, Julia Rzaska, Simon J. Foster, Michael A. Brockhurst, Alison M. Condliffe

**Affiliations:** a Department of Infection, Immunity and Cardiovascular Diseases, University of Sheffield, Sheffield, United Kingdom; b School of Biosciences, University of Sheffield, Sheffield, United Kingdom; c Florey Institute, University of Sheffield, Sheffield, United Kingdom; d Division of Evolution, Infection and Genomics, School of Biological Sciences, University of Manchester, Manchester, United Kingdom

**Keywords:** antibiotic resistance, evolution, hypoxia, *Staphylococcus aureus*, tetracycline, experimental evolution, doxycycline

## Abstract

Bacterial pathogens are confronted with a range of challenges at the site of infection, including exposure to antibiotic treatment and harsh physiological conditions, that can alter the fitness benefits and costs of acquiring antibiotic resistance. Here, we develop an experimental system to recapitulate resistance gene acquisition by Staphylococcus aureus and test how the subsequent evolution of the resistant bacterium is modulated by antibiotic treatment and oxygen levels, both of which are known to vary extensively at sites of infection. We show that acquiring tetracycline resistance was costly, reducing competitive growth against the isogenic strain without the resistance gene in the absence of the antibiotic, for S. aureus under hypoxic but not normoxic conditions. Treatment with tetracycline or doxycycline drove the emergence of enhanced resistance through mutations in an RluD-like protein-encoding gene and duplications of *tetL*, encoding the acquired tetracycline-specific efflux pump. In contrast, evolutionary adaptation by S. aureus to hypoxic conditions, which evolved in the absence of antibiotics through mutations affecting *gyrB*, was impeded by antibiotic treatment. Together, these data suggest that the horizontal acquisition of a new resistance mechanism is merely a starting point for the emergence of high-level resistance under antibiotic selection but that antibiotic treatment constrains pathogen adaptation to other important environmental selective forces such as hypoxia, which in turn could limit the survival of these highly resistant but poorly adapted genotypes after antibiotic treatment is ended.

## INTRODUCTION

Staphylococcus aureus is a Gram-positive commensal bacterium capable of causing a range of opportunistic infections (1). S. aureus has proven adept at acquiring resistance mechanisms, and its consequently increased level of antibiotic resistance poses a serious challenge to the effective treatment of infections (2, 3). Of particular concern is the ability of S. aureus to acquire novel resistance mechanisms by horizontal gene transfer (2). These include the tetracycline (TET)-specific efflux pumps Tet(K) and Tet(L), conferring resistance to multiple tetracyclines (4, 5). Tetracyclines are inexpensive broad-spectrum antibiotics widely used to treat both human and animal S. aureus infections globally. Doxycycline (DOX) is recommended for the treatment of outpatients with community-acquired pneumonia (6) and for many other respiratory and skin infections (7, 8), particularly in the context of penicillin allergy. Outpatient prescribing of doxycycline has recently increased with the onset of the coronavirus disease 2019 (COVID-19) pandemic, accounting for 10.5% of prescriptions in one study in 2020 compared to 4.9% in 2019 (9). Therefore, limiting the spread of tetracycline resistance is an urgent priority.

Horizontally acquired resistance mechanisms are unlikely to be well adapted to their new genomic context. Indeed, newly acquired resistance mechanisms are often associated with fitness costs (10, 11), which may in turn limit the survival and further spread of these resistance genotypes upon the removal of antibiotic selection (12). Such fitness costs can be due to a variety of causes, including the metabolic costs of transcribing and translating the resistance genes (13) or the incoming genes being mismatched with the new genomic environment (e.g., in terms of GC content or codon usage) or in conflict with other genes residing in the genome (11). Experimental evidence from a range of systems suggests that some of these initial fitness costs of acquiring resistance genes can be rapidly negated through compensatory evolution (14–16). Compensatory mutations may directly affect the newly acquired genes themselves or occur at other genomic loci (17) and have been shown to enable the maintenance of resistance genotypes both in the laboratory and in natural populations of bacteria (15, 18).

Variations in infection-relevant physiological parameters could also affect the fitness costs of newly acquired resistance mechanisms in pathogens. Conditions at infection sites can vary substantially, both between different types of infection and between spatiotemporal microenvironments within an infection (19, 20). Tissue oxygen levels are low, with oxygen tension falling from 100 mm Hg (13.3 kPa) in arterial blood to 20 to 30 mm Hg (3.6 to 3.9 kPa) in capillaries as oxygen diffuses into the surrounding tissues. This “physiological” hypoxia is further enhanced during infections due to the high respiratory demands of pathogens and immune cells, which result in marked oxygen consumption (21, 22). “Pathological” hypoxia is a common feature of S. aureus infections; for example, the archetypal S. aureus abscess can prevent oxygen exchange and antibiotic penetration through its fibrous capsule, exacerbated by the poor blood supply to the necrotic center (23, 24). S. aureus is exposed to hypoxia (or even anoxia) in many chronic infections, including the cystic fibrosis airway (25), chronic osteomyelitis (26), and diabetic foot ulcers (27), resulting in phenotypic changes to bacteria (28–30). S. aureus responds to a hypoxic environment through the two-component system *ssrAB* (staphylococcal respiratory response), regulating the production of virulence factors to resist oxidative stress and allowing metabolic adaptation (21, 31, 32). Additionally, short-term exposure to hypoxia *in vitro* has been shown to influence key pathogen characteristics, including biofilm formation and adhesion (33). However, the effect of hypoxia on the fitness costs of horizontally acquired resistance mechanisms, such as tetracycline efflux pumps, is currently unknown, although niche adaptation has been shown to enhance the stability of newly acquired resistance plasmids (34).

Antibiotic exposure can further alter the evolutionary response of bacteria to the horizontal acquisition of resistance genes. Exposing resistant cells to antibiotics can select for additional mutations affecting the horizontally acquired resistance mechanism itself and/or other genomic loci linked to resistance, potentially leading to the emergence of evolved genotypes with enhanced resistance (35, 36). For example, under tetracycline selection, populations of Escherichia coli carrying a plasmid encoding the TetA tetracycline-specific efflux pump evolved >32-fold-increased tetracycline resistance despite gaining mutations that impaired the activity of the TetA efflux system. Increased tetracycline resistance arose through compensatory missense mutations in chromosomal genes that reduced cellular permeability to tetracycline and increased its efflux (35). An S. aureus strain with a chromosomal copy of the methicillin resistance gene *mecA* evolved >2,000-fold-increased oxacillin resistance through the gain of missense mutations in *rpoB* or *rpoC*, encoding subunits of RNA polymerase, when selected on a gradient of oxacillin. The *rpo* mutations caused the increased expression of *mecA* and other chromosomal genes, causing enhanced resistance while also restoring the redox balance of the cell, which had been disrupted by the introduction of *mecA* (37).

Here, we develop an experimental evolution system to recapitulate the acquisition of a new antibiotic resistance mechanism in S. aureus, followed by an adaptation period, to test how these processes are affected by infection-relevant environmental conditions and treatment with TET or DOX. We show that the chromosomal acquisition of the tetracycline-specific efflux pump TetL caused a large fitness cost to S. aureus SH1000 under hypoxic but not normoxic conditions, demonstrating that the infection environment profoundly influences the fitness cost of this resistance mechanism. Contrasting evolutionary responses were observed after ~200 generations depending on the selective environment: S. aureus adapted to antibiotic treatments most frequently through mutations affecting an RluD-like protein, which increased resistance under both normoxia and hypoxia, and through duplication of *tetL*, encoding the tetracycline-specific efflux pump itself. In contrast, antibiotic treatment constrained S. aureus adaptation to hypoxia, which occurred mainly through mutations in *gyrB*, encoding DNA gyrase B, increasing fitness in hypoxic environments but only in the absence of antibiotics. Together, these data suggest that the horizontal acquisition of a new resistance mechanism is merely a starting point for the emergence of high-level resistance but that antibiotic treatment impedes pathogen adaptation to other key environmental selective forces such as hypoxia.

## RESULTS

### Tetracycline resistance is costly in hypoxia but not normoxia.

**FIG 1:**
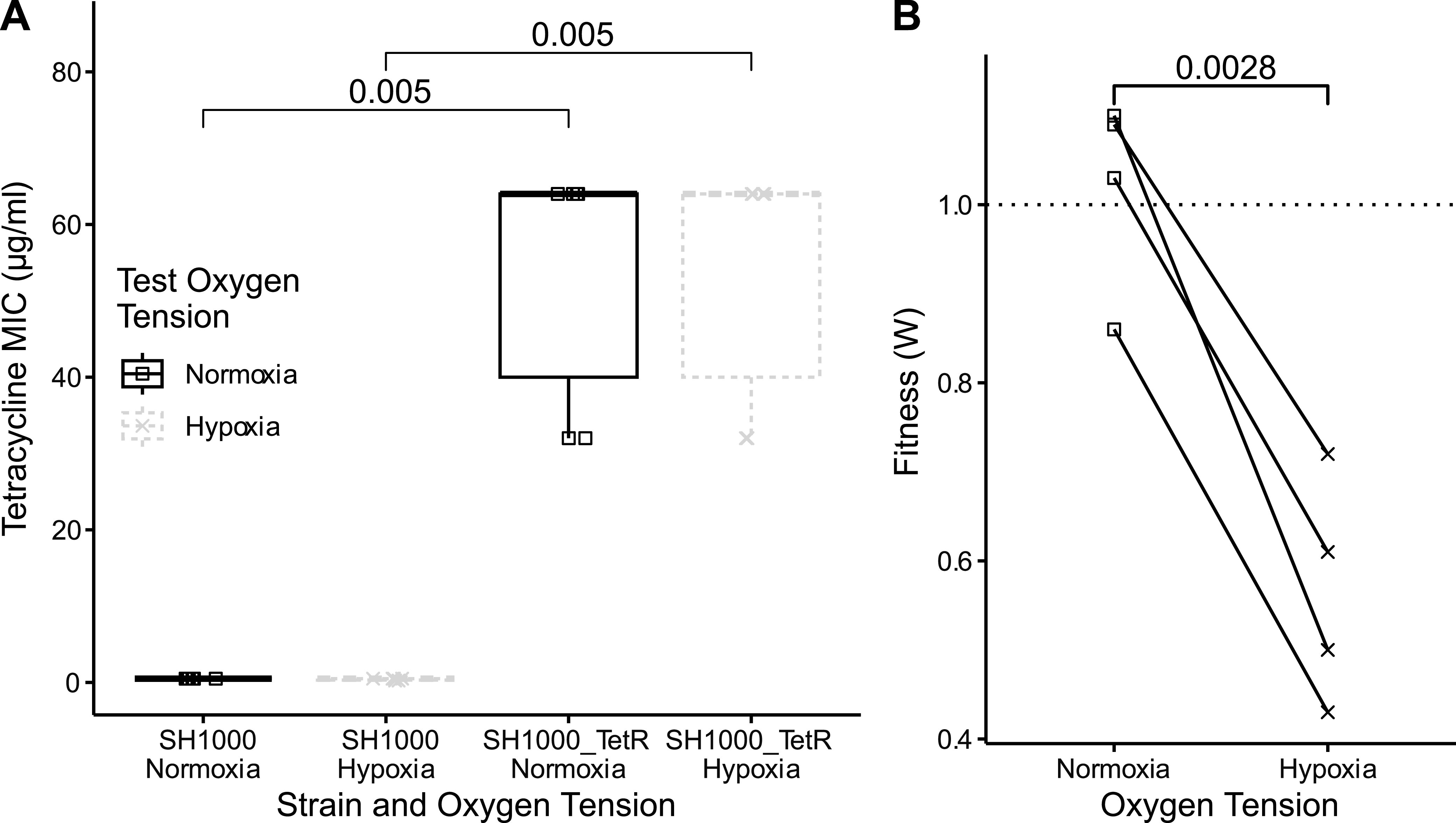
Effects of acquiring a tetracycline resistance gene on resistance and fitness in normoxia and hypoxia. (A) MICs of SH1000 and SH1000_TetR in TET prepared by serial dilutions of antibiotics in equilibrated BHI broth in normoxia or hypoxia in 96-well plates before a 20-h incubation with bacteria. MIC cutoffs were defined as the lowest antibiotic concentrations with no visible bacterial growth in the well (*n* = 6 independent experiments). Statistical analysis was performed by a Kruskal-Wallis rank sum test with Wilcoxon rank sum *post hoc* testing and Benjamini-Hochberg correction for multiple comparisons. (B) Competition assay showing the fitness of SH1000 competed against the isogenic strain with a *tetL* insertion at the *lysA* site (SH1000_TetR) in normoxia or hypoxia after 24 h of competition, starting at a 1:1 ratio. Results are from 4 independent experiments performed in triplicate, with lines connecting linked results. The dashed line at 1 indicates no fitness cost, and values of <1 show a fitness cost of SH1000_TetR. Analysis was performed by a paired *t* test.

To mimic the horizontal acquisition of a new antibiotic resistance gene, we used a S. aureus strain with the TET resistance gene *tetL* inserted into the SH1000 chromosome at the *lysA* locus (SH1000_TetR hereafter) (38). SH1000_TetR showed substantially increased resistance to TET and DOX relative to SH1000 in both hypoxia and normoxia (Fig. 1A) (normoxia, *P* = 0.005; hypoxia, *P* = 0.005). To test the immediate fitness effect of acquiring *tetL* and how this varied with the oxygen level, we competed SH1000_TetR against SH1000 under hypoxia (0.8% O_2_ and 5% CO_2_ at 37°C) and normoxia (21% O_2_ and 5% CO_2_ at 37°C) for 24 h. SH1000_TetR showed significantly lower fitness relative to SH1000 in hypoxia than in normoxia (Fig. 1B) (*P* = 0.0028), suggesting that *tetL* caused a large fitness cost but only under hypoxic conditions (normoxia, *P* = 0.74; hypoxia, *P* = 0.0064 [by one-sample *t* tests against 1]). Although the mechanism by which *tetL* causes this hypoxia-dependent fitness cost is unknown, the insertion of alternative resistance genes (*erm* or *kan*) at the same site did not cause differential fitness in hypoxia versus normoxia (SH1000_EryR, *P* = 0.823; SH1000_KanR, *P* = 0.837) (see Fig. S1 in the supplemental material), suggesting that the effect is specific to *tetL* and is not caused by disruption of *lysA*.

### Antibiotic treatment selected for increased resistance but no improvement in growth.

**FIG 2:**
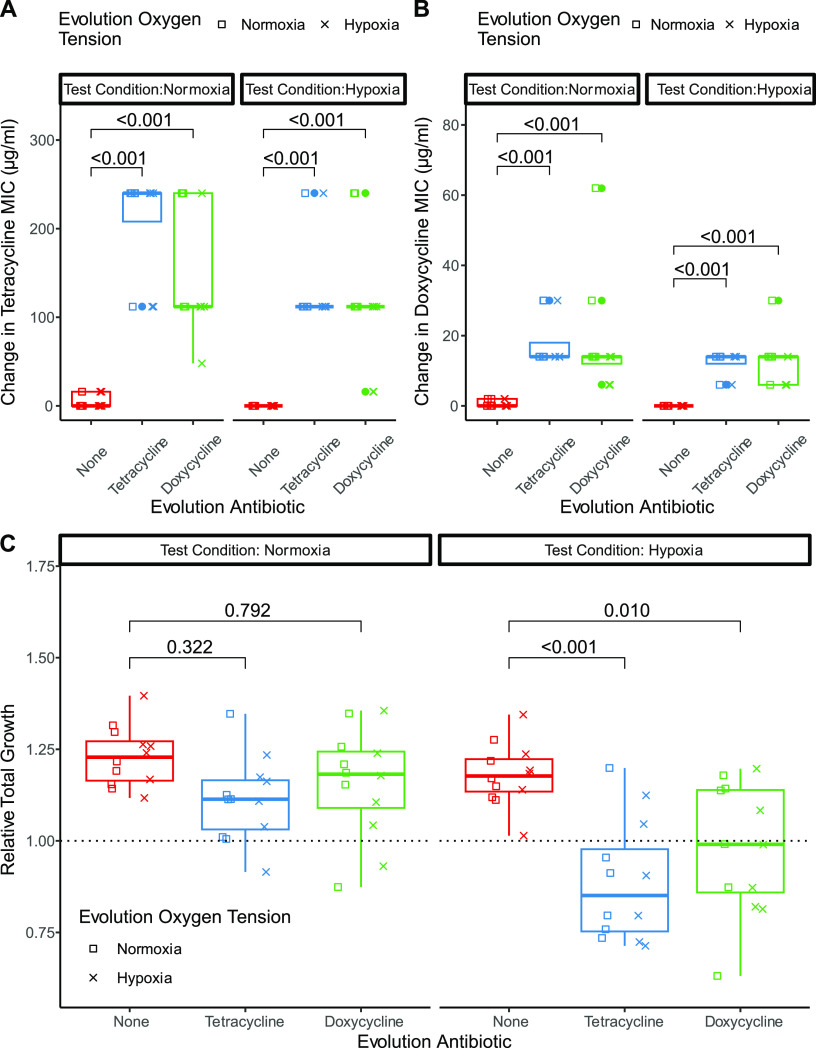
Resistance and growth phenotypes of the experimentally evolved populations. Population phenotypes of SH1000_TetR evolved in antibiotics and normoxia (squares) or hypoxia (crosses) relative to their ancestor are shown. Test oxygen conditions (normoxia or hypoxia) are indicated by panel labels. (A and B) Changes in the MICs compared to the ancestor measured in TET (A) or DOX (B), prepared as described in the methods section. Evolution antibiotics are represented in red for no antibiotic, blue for TET, and green for DOX. MIC cutoffs were defined as the lowest antibiotic concentrations with no visible bacterial growth in the well. Statistical analysis was performed by a Kruskal-Wallis rank sum test with Wilcoxon rank sum *post hoc* testing and Benjamini-Hochberg correction for multiple testing by evolution antibiotic, with no separation by evolution oxygen tension. A Kruskal-Wallis rank sum test was performed on all data independent of test oxygen tension by evolution antibiotic (*P* < 2.2 × 10^−16^ [A] and *P* < 2.2 × 10^−16^ [B]). (C) Relative integral of growth from evolved populations calculated from the growth curves of evolved SH1000_TetR populations grown in deep 96-well plates to match the evolution conditions. *A*_600nm_ readings were taken at 0, 2, 4, 6, and 24 h. The relative integral was calculated by division by the mean integral of the growth of the ancestor in matching test oxygen tensions (dashed line at 1). Results represent data from 3 experimental repeats. Statistical analysis was done with an ANOVA model [mean_integral ~ (evolution oxygen · evolution antibiotic · test oxygen tension)] with Tukey’s multiple-comparison test [evolution antibiotic, *P*r(>*F*) = 2.11 × 10^−5^ (by the ANOVA model)]. For full interactions, see Table S2 in the supplemental material.

To determine the longer-term evolutionary response to resistance gene acquisition and how this varied according to antibiotic selection and oxygen levels, we experimentally evolved replicate populations of SH1000_TetR with or without TET or DOX under either hypoxia or normoxia. Upon exposure to antibiotics, initially reduced population densities increased over time, reaching the level of those under antibiotic-free conditions. Recovery was stronger in populations selected under normoxia than in those selected under hypoxia, whereas the density of populations that evolved without antibiotics did not increase over time [time-antibiotic-oxygen tension interaction, *P*r(>*F*) = 0.0055] (Fig. S2 and Table S1). Correspondingly, antibiotic-treated populations evolved higher levels of antibiotic resistance such that populations treated with TET or DOX had higher levels of resistance against both antibiotics by the end of the experiment, whereas populations that evolved without antibiotics showed no change in resistance to either antibiotic (*P* < 4.3 × 10^−12^ by a Kruskal-Wallis rank sum test comparing the MICs of SH1000_TetR populations based on evolution with antibiotics) (Fig. 2A and B). For populations that evolved without antibiotics, significant albeit modest increases in both normoxic and hypoxic growth were observed by the end of the experiment (normoxic growth, *P* = 9.3 × 10^−7^; hypoxic growth, *P* = 1.5 × 10^−5^ [by a 1-sample *t* test against 1]). The hypoxic but not the normoxic growth of populations that evolved without antibiotics was significantly higher than that of populations that evolved with TET or DOX treatment {antibiotic, *P*r(>*F*) = 1.6 × 10^−5^ by the analysis of variance (ANOVA) model [normalized integral ~ (oxygen · antibiotic · condition)]} (Fig. 2C; for full interactions, see Table S2 in the supplemental material). Broadly consistent patterns of growth and resistance phenotypes were observed for a randomly chosen clone per population (later used for genome sequencing) (Fig. S3). Together, these data suggest that the horizontal acquisition of a tetracycline resistance gene came at a fitness cost apparent only under hypoxia and that this cost could be overcome through evolution only in the absence of antibiotics; antibiotics instead selected for higher levels of resistance.

### Distinct loci are selected by antibiotic- versus hypoxia-mediated selection.

**FIG 3:**
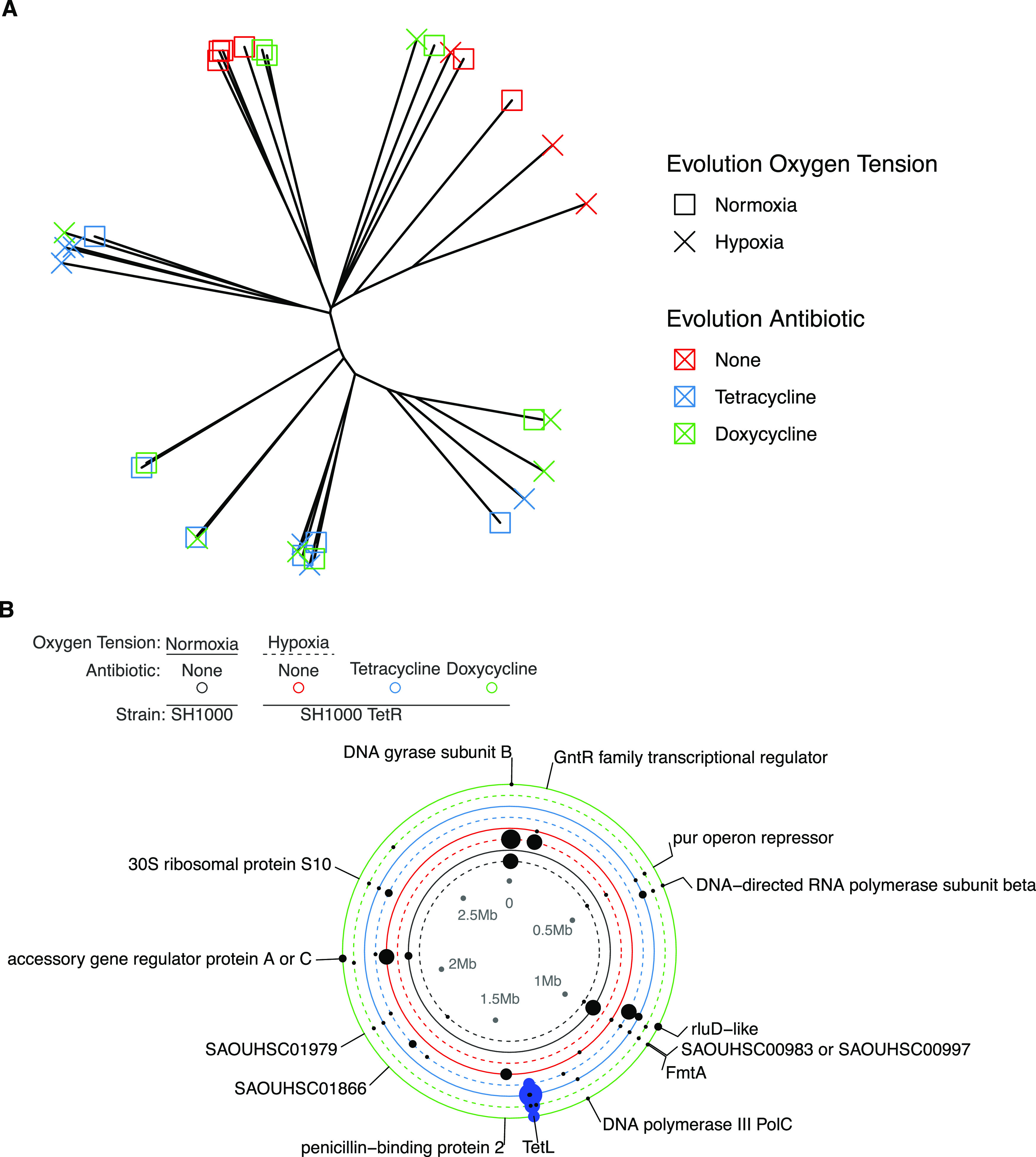
Genetic changes observed in the evolved clones. (A) Unrooted neighbor-joining phylogeny of SH1000_TetR clones. Distances are based on a matrix of nonsynonymous mutations in a binary change or no change in a gene, *tetL* duplication, recorded as duplicated or not. Evolution antibiotics are represented in red for no antibiotic, blue for TET, and green for DOX, with evolution oxygen tensions denoted with crosses for normoxia and squares for hypoxia (evolution antibiotics, *P* < 2 × 10^−16^; evolution oxygen tension, *P* = 1.84 × 10^−11^ [by the ANOVA model of Jaccard’s index of genetic distances based on selection conditions]) (no antibiotics versus TET, *P* < 1 × 10^−7^; no antibiotics versus DOX, *P* < 1 × 10^−7^; TET versus DOX, *P* = 0.133 [by Tukey’s multiple-comparison test]). (B) Nonsynonymous mutations (black) plus TetL duplication (blue) from day 30 evolved isolates. Synonymous mutations have been excluded for clarity. The sizes of the filled circles are proportional to the numbers of variants in different clones. Labels are for >1 variant in different clones. Black, SH1000 with no antibiotic; red, SH1000_TetR with no antibiotic; blue, SH1000_TetR with TET; green, SH1000_TetR with DOX. Evolution conditions are represented by solid lines for normoxia and dashed lines for hypoxia.

To investigate the genetic response to selection, we performed whole-genome sequencing on one randomly chosen clone per replicate population plus clones from control populations of SH1000 that had been evolved without antibiotics in either normoxia or hypoxia. A total of 115 mutations (Fig. 3; Fig. S4) were identified compared to their ancestor, comprising 8 deletions (6.9%), 7 frameshift mutations (6%), 31 intergenic mutations (27%), 7 synonymous mutations (6%), 48 missense mutations (41.7%), 11 nonsense mutations (9.5%), and 3 large deletions affecting more than 1 gene. There was no significant difference in the number of variants per clone between treatments [*P*r(>*F*) = 0.326 by 2-way ANOVA]. However, treatments varied in the loci that had acquired mutations [*F* = 7.427; *P*r(>*F*) = 0.001 (by permutational ANOVA)], with distinct sets of mutated loci associated with the response to antibiotic- or oxygen-mediated selection leading to genetic divergence between treatments (Fig. 3A) (antibiotics, *P* < 2 × 10^−16^; oxygen tension, *P* = 1.84 × 10^−11^).

To identify putatively adaptive mutations associated with the response to antibiotic and hypoxia selection, we focused on those loci that were mutated in multiple independently evolving populations within treatments (Fig. 3B) because such parallel evolution is strong evidence for the operation of positive selection at these loci (39). Although all evolved clones retained the *tetL* gene irrespective of treatment, in those that evolved with antibiotic selection, we observed a range of mutations at the site of the *tetL* insertion. Interrogation of the pMUTIN2 integration vector showed that 2/6 clones that evolved in DOX in normoxia had large deletions (of 995 bp in 2 sections in 1 clone and 7,659 bp in the other) in the pMUTIN2 gene after the *lysA* insertion site but retained the entire *tetL* gene. Elevated (>2-fold) coverage of the *tetL* gene was observed in 15 clones that had evolved under TET or DOX selection, suggesting gene duplication (Fig. S5A). The maximum coverage of *tetL* was 29× in a clone that had evolved under TET selection in normoxia. The location of the gene duplication was inspected, and no additional insertion sites were identified, suggesting that the gene duplications are within the *lysA* insertion site. Multiple genes involved in transcription and translation processes gained mutations in evolved clones selected with antibiotics. These included the 30S ribosomal protein S10 (5/47 clones all evolved in antibiotics), which is the target of tetracycline (40), and a gene encoding an RluD-like protein (10/47 clones all evolved in antibiotics), found in clones that evolved under both normoxia and hypoxia. Parallel mutations in genes encoding DNA gyrase B (10/47 clones [9 evolved in hypoxia]) and the GntR family transcriptional regulator (5/47 clones [4 evolved in hypoxia]) were observed more often in clones that evolved under hypoxia in the absence of antibiotics, suggesting that these are involved in the adaptation to hypoxia. Additional parallel mutations were identified in genes with no clear association with antibiotics or hypoxia, including penicillin-binding protein 2 (2/47 clones) and FmtA (10/47 clones), both of which are associated with cell wall synthesis (41, 42) and may therefore be associated with adaptation to laboratory conditions.

### Mutations affecting the RluD-like protein alter antibiotic resistance.

**FIG 4:**
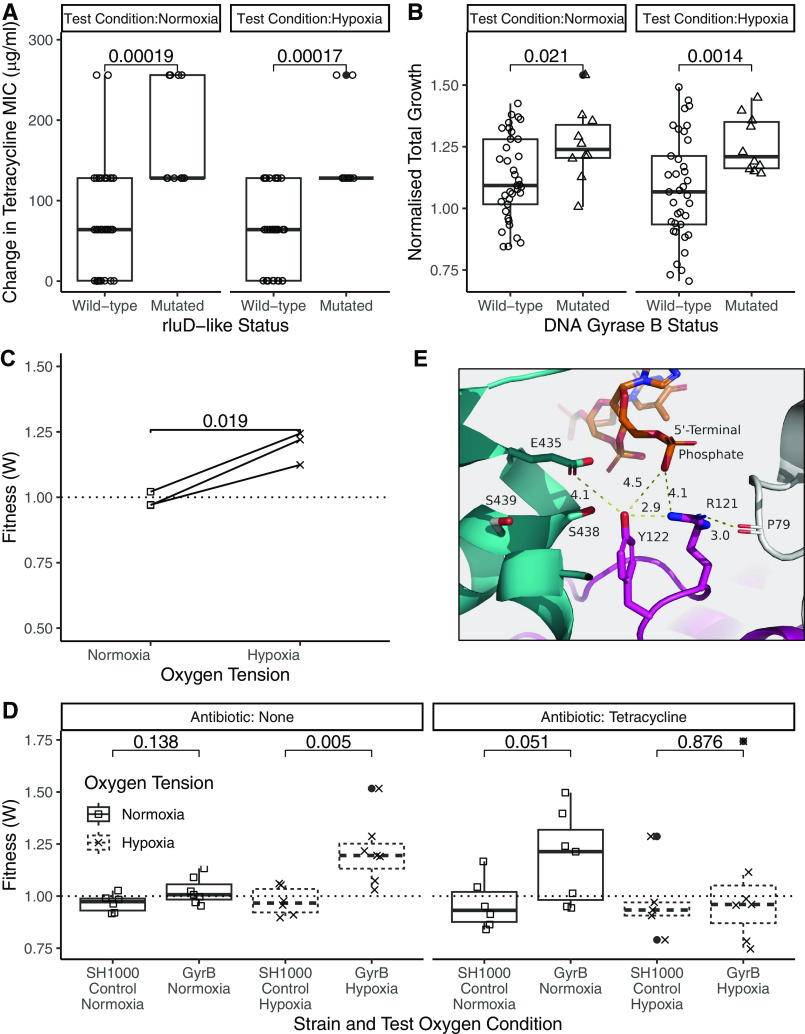
Effects of parallel mutations on the growth, resistance, and fitness of evolved clones. (A) MICs measured in TET prepared as described in the text prior to a 20-h incubation. MIC cutoffs were defined as the lowest antibiotic concentrations with no visible bacterial growth. Changes in the MICs of the sequenced clones compared to the ancestor are shown. The MICs of the sequenced clones with (*n* = 10) or without (*n* = 37) mutations in the *rluD*-like gene independent of mutations elsewhere in their genome are indicated. (B) Integrals calculated from the growth curves of clones from SH1000. Growth curves were carried out using deep 96-well plates to match the evolution conditions. *A*_600nm_ readings were taken at 0, 2, 4, 6, and 24 h. The relative integral was calculated by division by the mean integral of the growth of the ancestor in matching test oxygen tensions. Clones without DNA gyrase B mutations (*n* = 37) were compared to variants with DNA gyrase B mutations (*n* = 10) independent of whether the clones carried other mutations within their genomes. (C and D) Competition assays of S. aureus competed against SH1000_KanR for 24 h in normoxia or hypoxia. Starting and final ratios were determined by selective plating with or without kanamycin. The dashed line at 1 indicates no fitness cost. Values of <1 show a fitness advantage compared to SH1000_KanR. (C) Three clones carrying the A439S variant in DNA gyrase B and no other variants. Results are from experiments with 3 clones carried out in triplicate, with a line connecting the same clone. (D) Ancestor SH1000 compared to 2 clones (*n* = 3/4) carrying the A439S variant and no other mutations. TET is included in the competitions for the 24-h incubation at 0.125 μg/mL. Results are from 7 experiments carried out in triplicate. (E) Computational structural analysis of the A439S DNA gyrase B mutation. Ser439 mutations increase H bond networks and create a pocket possibly suitable for an H_2_O molecule or ion, which could enhance the H bond network and phosphotyrosine DNA interactions. Cyan, chain B; pink, chain C; gray, chain A; orange, DNA. All distances are measured in angstroms. Statistical analysis was performed by a Kruskal-Wallis rank sum test with Wilcoxon rank sum *post hoc* testing and Benjamini-Hochberg correction for multiple comparisons (A, B, and D) or a paired *t* test (C).

To associate the observed genetic variants with specific phenotypes, the tetracycline MICs and the growth of clones with and without mutations at loci of interest were compared. Mutations in the gene encoding an RluD-like protein were associated with a significant increase in the tetracycline MIC when tested in both normoxia and hypoxia (Fig. 4A) (normoxia, *P* = 0.00019; hypoxia, *P* = 0.00017). The poorly characterized *rluD*-like protein is a pseudouridine synthase that carries out posttranscriptional modification of 23S rRNA (43). Ten clones carried a mutation within the *rluD*-like gene, all of which had evolved in the presence of antibiotics. These variants were in 5 different positions in the gene. Residue 29 was mutated in 4 clones, with 3 clones having the A29T variant and 1 having the A29S variant. All clones carrying an *rluD*-like variant also had 1 or more mutations elsewhere in their genomes compared to their ancestor.

Another genetic variant favored by antibiotic selection was the *tetL* duplication (15/24 clones evolved in TET or DOX) (Fig. S5A). Two such clones had no mutations elsewhere in their genomes, both of which evolved in DOX in either normoxia (15.6× coverage) or hypoxia (26.2× coverage). Competing evolved clones with *tetL* duplications but no other mutations showed no change in fitness compared to the SH1000_TetR ancestor with or without tetracycline, irrespective of oxygenation (antibiotic free, *P* = 0.932 for normoxia and *P* = 0.989 for hypoxia; TET, *P* = 0.99 for normoxia and *P* = 0.763 for hypoxia) (Fig. S5B), suggesting that the *tetL* duplications did not lead to a higher fitness cost. There was no clear association between the increase in the *tetL* coverage of clones and their tetracycline MICs (Fig. S5C); 3/8 clones had the maximum TET MIC of 256 μg/mL without *tetL* duplication. The increased resistance of these clones was instead associated with a combination of mutations in genes encoding the RluD-like protein (2 clones), accessory gene regulator protein A (1 clone), RpoB (1 clone), 30S ribosomal protein S10 (1 clone), a hypothetical protein (1 clone), and a 7,659-bp deletion in the *tetL* insertion cassette (1 clone [resistance gene intact]). These achieve the same high-level tetracycline resistance, thus obscuring any benefit of *tetL* duplication and suggesting that chromosomal point mutations elsewhere in the genome contribute to the high-level resistance. In addition, two highly TET-resistant evolved clones from different treatments carried a parallel nucleotide mutation in a noncoding region between *malK* and *lytM* (2 clones without *tetL* duplications, including 1 with this as the only mutation). However, an identical mutation was present in all of the replicate 2 sequenced SH1000_TetR evolved clones regardless of the treatment, suggesting that this mutation was present in the ancestral clone used to found these replicate lines (Fig. S4).

### DNA gyrase B mutation increases S. aureus fitness in hypoxia.

Evolved clones with mutations in *gyrB* showed increased growth in both normoxia (*P* = 0.021) and hypoxia (*P* = 0.0014) (Fig. 4B). DNA gyrase B is a topoisomerase that catalyzes the negative supercoiling of double-stranded DNA (44). Overall, 10 clones (SH1000 or SH1000_TetR lineage) carried a DNA gyrase B variant, 9 of which were evolved under hypoxia with no antibiotics and 1 of which was evolved under normoxia with DOX. Four clones carried the A439S variant, 3 of which had no additional mutations. Competition assays of these clones against their ancestor showed significantly increased fitness relative to the ancestor in hypoxia compared to normoxia (*P* = 0.019) (Fig. 4C). The strong association of *gyrB* mutations with the antibiotic-free environment was interrogated through competition assays performed with or without tetracycline (0.125 μg/mL). Clones with the DNA gyrase B A439S variant showed increased fitness under antibiotic-free hypoxic conditions compared to their ancestor (Fig. 4D) (hypoxia, *P* = 0.005) but not under normoxia (*P* = 0.138) or in the presence of TET (normoxia, *P* = 0.051; hypoxia, *P* = 0.876). These data support the increased fitness under antibiotic-free conditions in hypoxia driving the selection of DNA gyrase B mutations, with the addition of antibiotics in hypoxia eliminating this fitness advantage.

To better understand the role of *gyrB* mutations, we performed a computational structural analysis of the DNA gyrase B A439S variant protein. Residue 439 lies within a relatively hydrophobic pocket in the active site; thus, an alanine-to-serine mutation could be making it more hydrophilic. When including the DNA chain within the structure, the change to Ser439 (subunit B) is in proximity to Tyr122 (subunit A), which forms a phosphotyrosine interaction with the DNA. The space between Ser439 and Tyr122 creates a space and appropriate bonding for an H_2_O molecule or ion (Fig. 4E; Fig. S6), which could create a network of H bonds to stabilize the active site affecting DNA binding. As such, this mutation may alter the function of DNA gyrase B.

## DISCUSSION

Bacterial pathogens encounter various challenges at the site of infection, including exposure to antibiotic treatments and physiological stresses (11, 23, 45). Such selective environments are likely to modulate the costs and benefits of acquiring new resistance determinants and, hence, alter the longer-term evolutionary trajectory of antibiotic-resistant lineages. Here, we show that hypoxia, a common physiological stress at infection sites (21, 30), increases the cost of acquiring the TetL tetracycline efflux pump in S. aureus. Prolonged antibiotic treatment drove the evolution of increased resistance to tetracycline and doxycycline via mutations affecting an *rluD*-like gene or through duplication of *tetL* but constrained adaptation to hypoxia, which otherwise arose via mutations affecting *gyrB*. Crucially, the fitness benefits of *gyrB* mutations under hypoxic conditions were entirely negated in the presence of tetracycline.

The horizontal acquisition of new resistance genes is often expected to be associated with fitness costs (10, 11), which could limit the persistence of resistance genotypes in the absence of antibiotics. Our data demonstrate that such fitness costs depend critically upon environmental oxygen availability, with a high cost of acquiring a tetracycline efflux pump observed under hypoxia but not normoxia. This suggests that measurements made under standard normoxic laboratory conditions may underestimate the fitness costs of tetracycline resistance. Hypoxia is common in a range of infection environments, with oxygen tensions of 20 to 30 mm Hg in normal capillaries (21) and 2.5 mm Hg in the mucus of patients with cystic fibrosis (30). The high cost of tetracycline resistance under hypoxia may therefore help to explain why tetracyclines continue to be useful treatments against S. aureus infections within such hypoxic infection environments.

In the absence of antibiotics, S. aureus adapted to hypoxia via mutations affecting *gyrB*. DNA gyrase B is the second subunit of the gyrase type II topoisomerase responsible for simultaneously catalyzing the breakage and formation of double-stranded DNA and removing negatively supercoiled DNA in the absence of ATP. As such, DNA gyrase has broad influences on bacterial cell processes, including DNA replication, transcription, recombination, and repair (44, 46). Importantly, DNA gyrase has been shown to be essential for anaerobic but not aerobic growth (47), while increases in supercoiling have been associated with short-term transitions to anaerobic conditions. A *gyrB226* mutation in E. coli has previously been shown to decrease supercoiling associated with a shift to anaerobic conditions (48). We hypothesize, therefore, that the mutations that we observed in DNA gyrase B may have increased bacterial fitness in hypoxia through modulating negative supercoiling and the associated regulation of gene expression. By increasing fitness in low-oxygen environments, *gyrB* mutations may enhance the competitiveness and survival of S. aureus at hypoxic infection sites. Changes in the rates of transcription may support adaptation to hypoxia in *gyrB* mutants and variants with mutations affecting a GntR family transcription regulator, which were also more prevalent in hypoxia. These findings thus suggest a previously unknown regulatory mechanism for adapting to hypoxia. Somewhat unexpectedly, we did not observe mutations in genes previously associated with the response to hypoxia, such as *srrAB* encoding a 2-component system (31), although we cannot rule out the possibility that changes in gene expression levels could be occurring in parallel.

In common with other studies (35), we observed that under prolonged antibiotic selection, resistance genotypes evolve to further increase their resistance despite already possessing an adequate resistance mechanism. Increased resistance to tetracycline and doxycycline arose via two distinct mechanisms. First, we observed duplication of *tetL*, the gene encoding the tetracycline efflux pump. Gene duplication events are commonly observed under antibiotic selection *in vitro* (49), including the duplication of plasmid-borne *tetL* in Enterococcus faecalis (50), which was reversed upon the removal of tetracycline. Gene duplications mediating antibiotic resistance have also been detected in S. aureus isolated from clinical infections (51, 52). Resistance gene duplication events that increase resistance are likely to be more common than observed because these genomic changes are typically rapidly reversed in cultures without antibiotics (49–51), although we did not test this in our evolved clones. The *tetL* duplication events did not incur an additional fitness cost, which is in line with the results of previous studies, although we cannot rule out a loss of gene duplications during the competition assay (49).

A second mutational mechanism leading to increased antibiotic resistance was through mutations affecting an RluD-like protein, which were strongly associated with increased tetracycline resistance. The RluD-like protein is a pseudouridine synthase that carries out posttranscriptional modifications of 23S rRNA (43) and therefore is associated with the target of tetracyclines, the 30S ribosomal protein. The potential clinical relevance of these mutations is supported by the identification of RluD-like protein mutations (A29T) in a clinical isolate (53). Mutations within the RluD-like protein have been observed in a previous laboratory evolution experiment wherein the same S. aureus SH1000 strain was selected in the presence of the antimicrobial peptide melittin (54). All 3 melittin-resistant strains identified in that study had mutations of residue 35 of the RluD-like protein, as did 1/10 RluD-like variants identified in our study. However, the precise mechanism of resistance against tetracycline versus melittin is likely to differ due to the contrasting mode of action of the antimicrobial peptide, which targets the bacterial membrane, resulting in cell lysis (55).

Although we used hypoxia as an infection-relevant selection pressure, a limitation of our study is that our experimental conditions do not fully capture the complexity of the infection environment. Conditions at infection sites are likely to vary with time and include additional stressors such as acidosis, nutrient deprivation, immune factors, and interactions with other microbes in mixed infections (23, 24, 45). While some studies have explored the influence of fluctuating conditions (35, 56), more studies incorporating a range of host-specific stresses are needed to understand both the mechanisms of pathogen adaptation to these conditions and how such conditions affect the fitness costs of acquiring antibiotic resistance genes.

Hypoxia is common in many S. aureus infections, and our results highlight the potential for such host-relevant physiological stresses to interact with antibiotic selection in complex and unpredictable ways. By modulating fitness costs, hypoxia may select against antibiotic resistance, whereas antibiotic selection may constrain adaptation to hypoxic conditions, thus potentially limiting the persistence of these highly resistant but poorly adapted genotypes once antibiotic treatment ceases. Together, these combined effects may help to explain why tetracyclines remain a useful treatment against S. aureus infections where hypoxia is a prominent feature of the within-host environment.

## MATERIALS AND METHODS

### Bacterial strains, culture, and controlling oxygen tensions.

All strains used in this study are listed in Table S3 in the supplemental material. Bacteria were cultured in brain heart infusion (BHI) broth (280 rpm) or BHI agar. Hypoxia was maintained in a Ruskinn SCI-tive chamber (0.8% O_2_, 5% CO_2_, and 70% humidity at 37°C) with all experiments matched in normoxia (21% O_2_, 5% CO_2_, and controlled humidity at 37°C) and medium equilibrated overnight (280 rpm) before use. Cultures were grown overnight in normoxia before use with either oxygen tension.

### Competition assays.

Strains were grown overnight in 10 mL BHI broth in normoxia to stationary phase. Fifty microliters (~5 × 10^5^ CFU) of each competing strain was inoculated at a 1:1 ratio into 10 mL equilibrated BHI broth under normoxia or hypoxia before growth for 24 h, with or without 0.125 μg/mL tetracycline (TET). The starting and final bacterial densities were calculated by plating a serial dilution onto BHI agar with or without TET (5 μg/mL) or kanamycin (5 μg/mL), depending on the resistant cassette in the competing strain. Fitness (*W*) was calculated as *W* = ln[(*A* end CFU/mL)/(*A* start CFU/mL)]/ln[(*B* end CFU/mL)/(*B* start CFU/mL)].

### Selection experiment.

To establish experimental lines, 6 independent colonies for SH1000 controls and SH1000_TetR were selected. Individual colonies were inoculated into 10 mL BHI broth and grown in normoxia overnight, known as populations 1 to 6. Cultures grown overnight at 1:100 dilutions started 8 experimental lines under the treatment conditions (antibiotic-free SH1000_TetR, SH1000_TetR with 30 μg/mL TET, and SH1000_TetR with 2 μg/mL DOX) plus the controls (antibiotic-free SH1000), all matched in normoxia and hypoxia. Cultures for all treatments were grown in a final volume of 400 μL BHI broth in 1.2-mL 96-well plates sealed with gas permeable film to allow gas exchange while preventing contamination. Every 24 h for 34 days, each population had a 1:100 serial passage into fresh BHI broth, and the *A*_600nm_ was measured. Twenty percent cryogenic glycerol stocks of whole populations were prepared and stored at −80°C every 5 transfers, and a subsection was plated. Population 6 of SH1000 in normoxia died out on day 14 and was excluded from subsequent analyses.

### Growth curves.

Cultures were grown overnight directly from a 20% glycerol stock in normoxia prior to inoculation at a mean 1:100 dilution of the populations grown overnight into 400 μL preequilibrated BHI broth in deep-well 96-well plates with slitted plate seals in normoxia or hypoxia, including the ancestor controls. These cultures were grown at 280 rpm for 0, 2, 4, 6, or 24 h, at which time the optical density at 600 nm (OD_600nm_) was measured by destructive sampling with a separate plate at each time point. The growth of the evolved population or clone was compared to that of the ancestors grown simultaneously under identical conditions.

### MIC assays.

Populations grown overnight from 20% glycerol stocks were diluted to an *A*_600nm_ of 0.05 in BHI broth. Ten microliters of these cultures was added to 90 μL of antibiotics prepared in a 96-well plate with log_2_ serial dilutions prepared in preequilibrated BHI broth in normoxia or hypoxia. The final 100-μL cultures were grown for 20 h in normoxia or hypoxia before measurement of the OD_600nm_. MIC cutoffs were determined as the lowest concentration of the antibiotic without visible growth.

### Genome sequencing and bioinformatic analysis.

A single colony of each evolved population was randomly selected before short-read sequencing was performed by MicrobesNG (www.microbesng.com) using an Illumina MiSeq platform with 2× 250-bp paired-end reads. Sequence analysis was performed according to methods described previously by Wright et al. (57) against the reference strain NCTC 8325 (GenBank genome accession number CP000253). Larger genetic variations, including deletions of >100 bp and duplication events, were identified by analyzing changes in the coverage depth using R version 4.0.3, and all variants were verified visually using IGV (58). The Breseq pipeline (59) was used to check for additional variants.

The *tetL* insertion was interrogated through the extraction of unmapped reads before *de novo* assembly using SPAdes (60). Mutations were checked using the sequence analysis described above, with the SH1000_TetR SPAdes assembly of the resistance gene as the reference. Changes in *tetL* coverage were determined by comparison of the mean coverage within the *tetL* gene to the mean coverage of the corresponding genome. Additional insertion sites were checked by the extraction and sorting of unmapped reads (from the initial pipeline) where at least 1 read aligns to the SH1000_TetR SPAdes assembly using the Burrows-Wheeler aligner (61), followed by SPAdes assembly to compile contigs to identify insertion sites by NCBI BLAST (62).

### Mutation structural analysis.

Structural predictions were carried out using Phyre2 (63) with the resulting structures analyzed using PyMOL (64) to structurally align them to the cryo-electron microscopy (CryoEM) structure reported for E. coli DNA gyrase under Protein Data Bank (PDB) accession number 6RKW (65).

### Statistical analysis.

All statistical analyses were carried out using R version 4.0.3. To test for interactions between evolution time, antibiotic exposure, and oxygen tension, a linear mixed-effects model (R package lme4) was used on the data with population number as a random effect following Box-Cox transformation to meet the underlying test assumptions. Phylogenetic distances were calculated using the Jaccard index based on the presence or absence of mutations within a gene or *tetL* duplication followed by permutational ANOVA (vegan package). Prior to statistical analysis, the normality of data was tested with a Shapiro-Wilk test. Statistical analysis of parametric data was carried out by ANOVA with Tukey’s multiple-comparison test, a paired *t* test for 2 groups of paired data, a *t* test for 2 groups of unpaired data, and an analysis of variance model with antibiotic and evolution oxygen tension as independent variables followed by Tukey’s multiple-comparison test of evolution phenotype data. Nonparametric data were analyzed by a Kruskal-Wallis rank sum test with Wilcoxon rank sum *post hoc* testing and Benjamini-Hochberg correction for multiple comparisons (66).

### Data availability.

All sequencing data are available in the Sequence Read Archive under BioProject accession number PRJNA850453. All study data are included in the supplemental material.
