# Supplementary material for: Antibiotics Limit Adaptation of Drug-Resistant Staphylococcus aureus to Hypoxia
Source: Antimicrob Agents Chemother. 2022 Nov 21;66(12):e00926-22. doi: 10.1128/aac.00926-22 (PMC9765076; doi:10.1128/aac.00926-22)
Supplement: Supplemental file 2 — Fig. S1 to S6 and Tables S1 to S3. Download aac.00926-22-s0002.pdf, PDF file, 0.6 MB [file aac.00926-22-s0002.pdf]

Supplementary Material

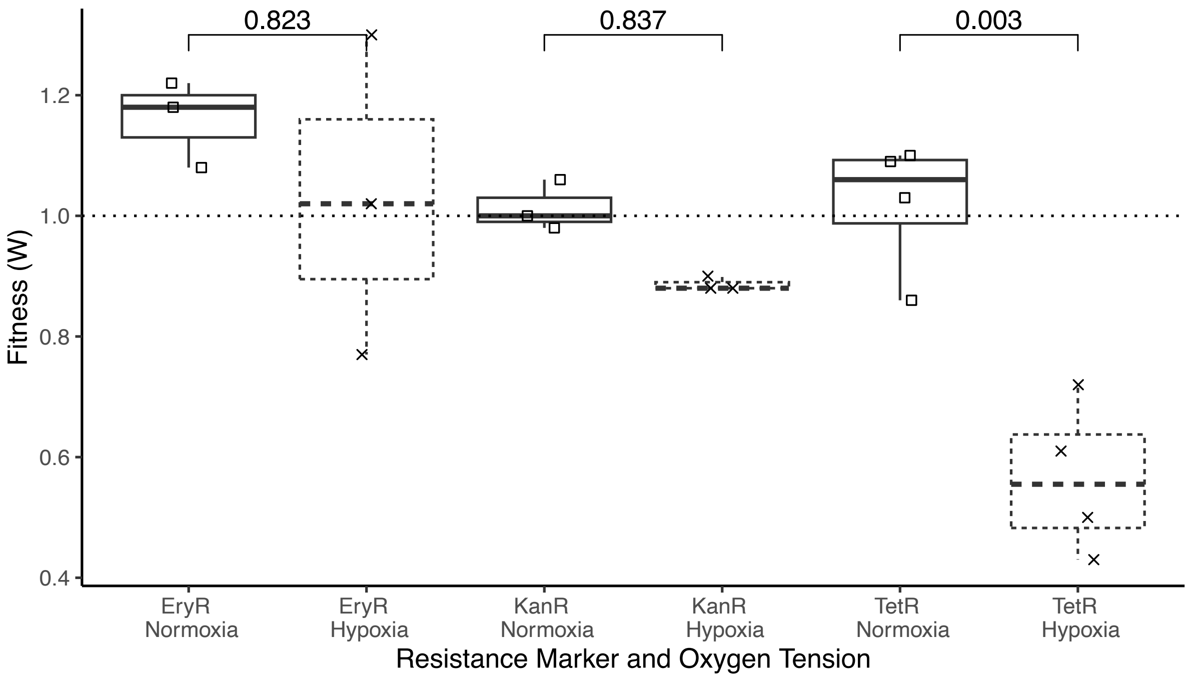

Supplementary Figure S1: Fitness effect of insertion of erythromycin, kanamycin and tetracycline resistance gene into SH1000 under normoxia and hypoxia

Relative fitness of SH1000 with EryR, KanR or TetL insertion at LysA site (SH1000_EryR, SH1000_KanR, SH1000_TetR) relative to isogenic SH1000 in normoxia or hypoxia after 24h direct competition, starting from a 1:1 ratio. Showing results from n=3/4 independent experiments each performed in triplicate. A Fitness value of 1 indicates equal fitness as indicated by the dashed line. Analysis by ANOVA with Tukey's multiple comparisons. SH1000_TetR data is also shown in Figure 1.

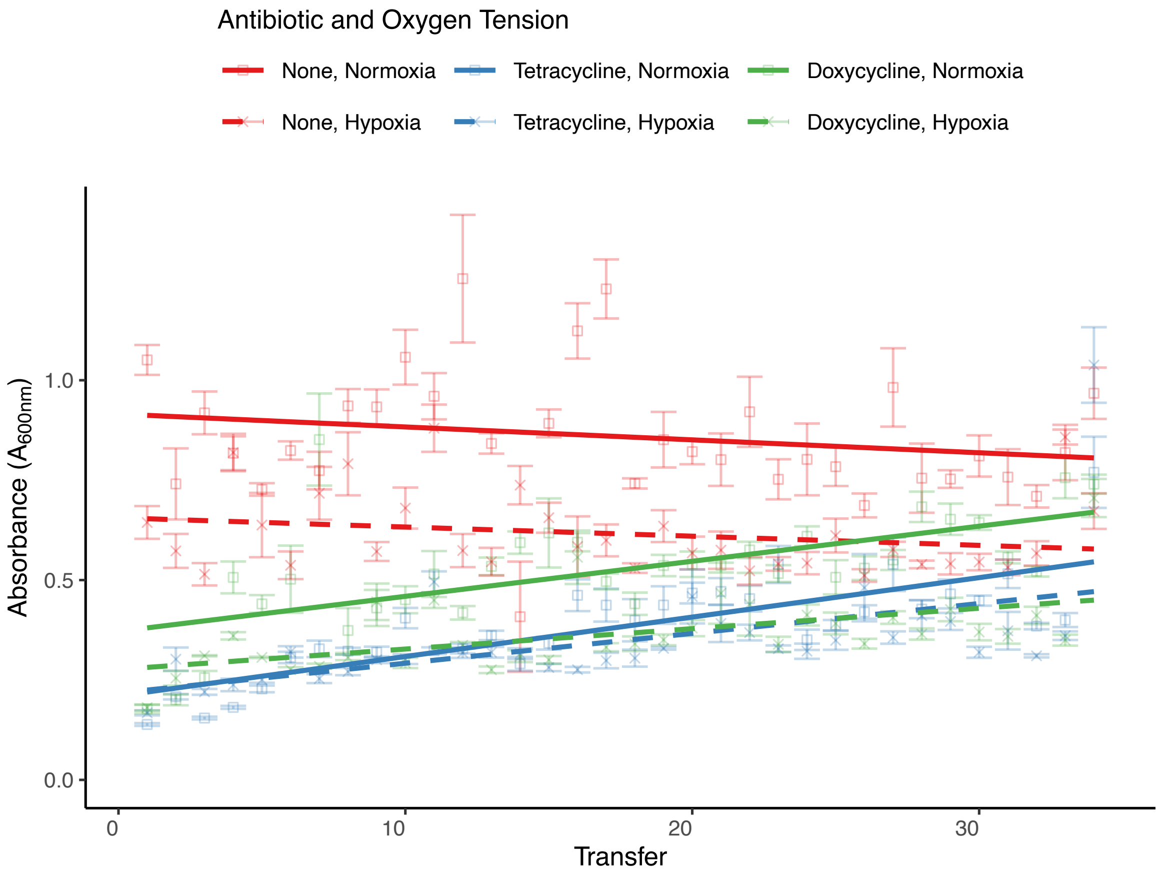

Supplementary Figure 2: Population densities of evolution treatments over time.

Population densities quantified as OD_{600} readings of each evolving population over time. Readings were taken after 24h growth in pre-equilibrated BHI broth in normoxia (solid line, square) (21% O_2, 5% CO_2) or hypoxia (dashed line, cross) (0.8% O_2, 5% CO_2) prior to serial transfer. Antibiotic treatments are indicated by colours: Red = antibiotic free, blue = TET (30µg/ml), green = DOX (2µg/ml). Datapoints show mean of n=6 populations ± standard error bars show. Trend lines show fitted linear model. Full statistical table given in supplementary Table 1.

Supplementary Table 1: Linear mixed effects model output.

*Type III Analysis of Variance Table with Satterthwaite's method of linear mixed effects model from Supplementary Figure 2. Signif. codes: 0 '***' 0.001 '**'.*

Interaction	Sum Sq	Mean Sq	NumDF	DenDF	F value	Pr(F)	
Transfer	32.43	32.43	1	1801.7	307.52	< 2.2e-16	***
Oxygen Tension	0.92	0.92	1	1801.1	8.72	0.0032	**
Antibiotic	134.19	67.09	2	1801.3	636.15	< 2.2e-16	***
Transfer: Oxygen Tension	3.98	3.98	1	1801.1	37.71	1.003e-09	***
Transfer: Antibiotic	26.72	13.36	2	1801.7	126.68	< 2.2e-16	***
Oxygen Tension: Antibiotic	4.28	2.14	2	1801.1	20.28	1.950e-09	***
Transfer: Oxygen Tension: Antibiotic	1.10	0.55	2	1801.1	5.22	0.0055	**

Supplementary Table 2**: **ANOVA model output. *ANOVA model of integral growth curves of evolved populations in Figure 2C. Model: normalised integral ~ (Evolution Oxygen*Evolution Antibiotic*Test Oxygen Tension)*

Interaction	DF	Sum Sq	Mean Sq	F value	Pr(F)	
Evolution Oxygen	1	0.012	0.0012	0.060	0.0868	
Evolution Antibiotic	2	0.5214	0.2607	13.313	1.64×10^{-5}	***
Test Oxygen	1	0.4104	0.4104	20.958	2.42×10^{-5}	***
Evolution Oxygen: Evolution Antibiotic	2	0.0063	0.0031	0.160	0.8521	
Evolution Oxygen: Test Oxygen	1	0.0000	0.0000	0.001	0.9821	
Evolution Antibiotic: Test Oxygen	2	0.0971	0.0485	2.479	0.0924	
Evolution Oxygen: Evolution Antibiotic: Test Oxygen	2	0.0002	0.0001	0.005	0.9951	
Residuals	60	1.1749	0.0196			

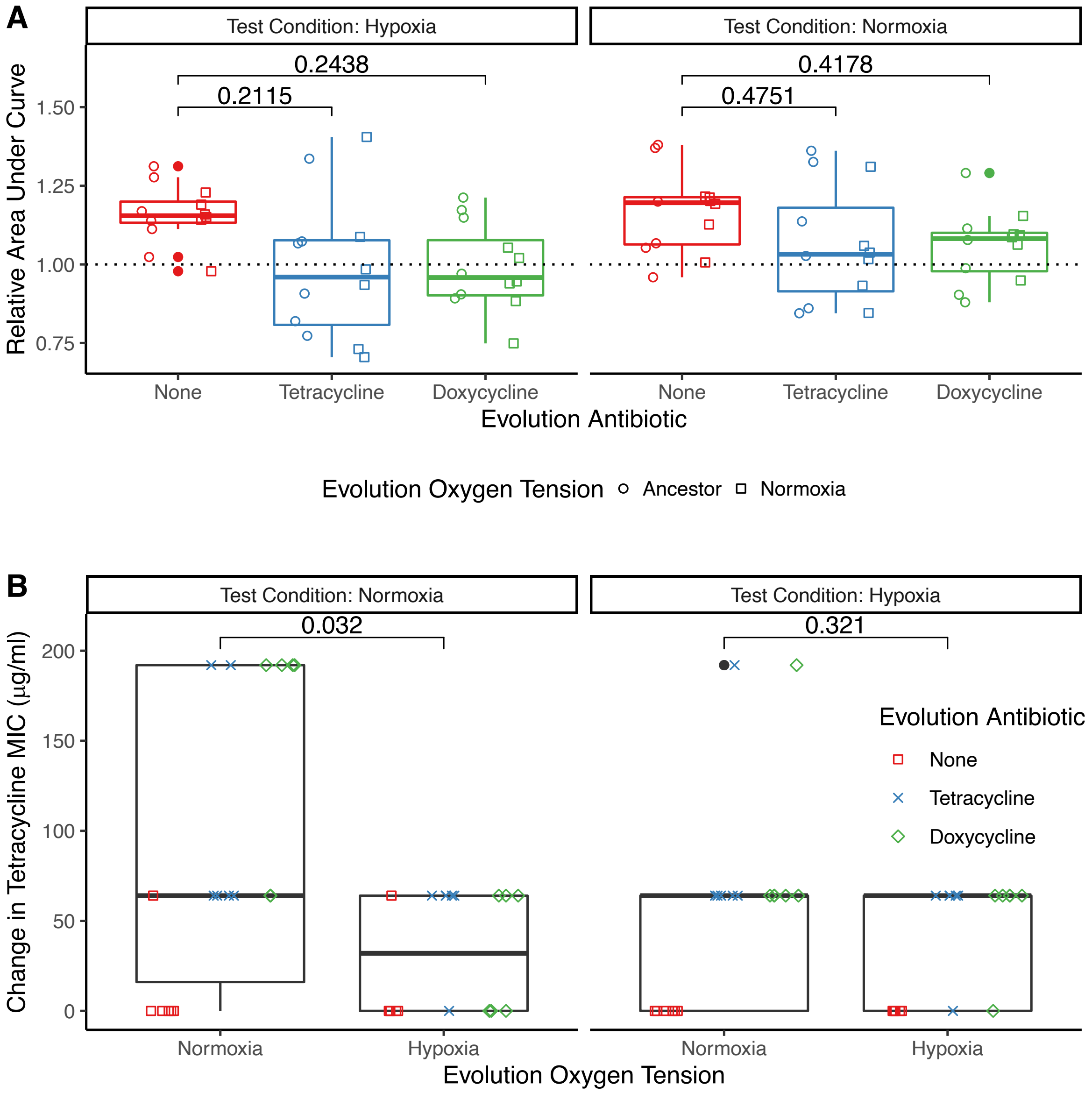

Supplementary Figure 3: Minimum inhibitory concentrations and growth metrics of evolved clones.

A) Integral calculated from growth curves of clones from SH1000. Growth curves were carried out in deep 96 well plates in to match evolution condition. $0D_{600}$ readings were taken at 0,2,4,6,24h. The relative integral was calculated by division by the mean integral of the ancestor growth in matching test oxygen tensions. n=5/6 clones evolved from SH1000 in each evolution condition. Statistical analysis by ANOVA

*model (mean integral ~ (Evolution Oxygen*Evolution Antibiotic*Test Oxygen Tension) with Tukeys multiple comparisons. ANOVA model, evolution antibiotic, Pr(>F)= 0.00584. B) MICs measured in TET prepared by serial dilutions of antibiotics in equilibrated BHI broth in normoxia or hypoxia in 96 well plates. Bacteria were grown overnight before dilution to OD_{600}=0.05 before adding to TET dilutions. Following 20h incubation MIC cut-offs were defined as the lowest antibiotic concentration with no visible bacterial growth. n=6 clones evolved from SH1000_TetR in each evolution condition. Change in MIC calculated by deducting ancestors MIC. Statistical analysis Kruskal-Wallis rank sum test with Wilcoxon rank sum post-hoc testing and Benjamini-Hochberg correction for multiple comparisons. Kruskal-Wallis rank sum test independent of test oxygen tension evolution oxygen hypoxia: normoxia, p=0.022.*

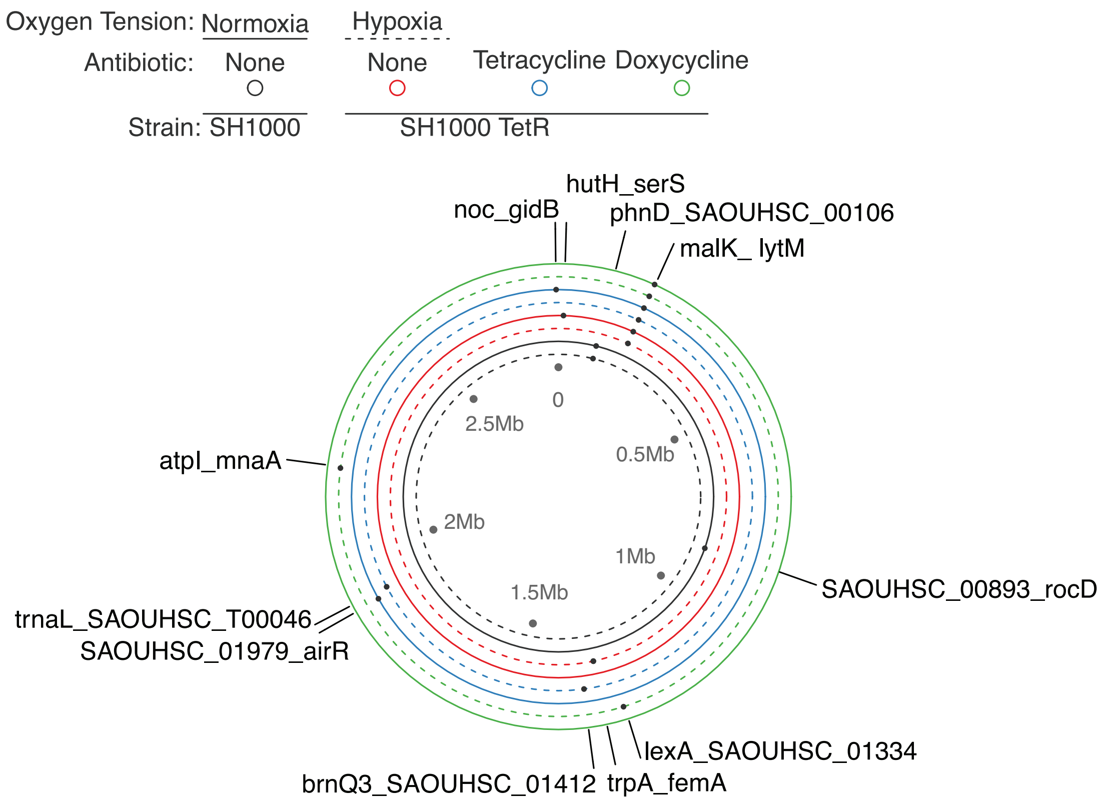

Supplementary Figure 4: Intergenic mutations

Intergenic (black) mutations from day 30 evolved isolates. Concentric circles represent the genomes of evolved clones. Filled circles represent an intergenic mutation at that position and labels denote the adjacent genes. Evolution conditions are represented by colours and line type: Black = SH1000 no antibiotic, red = SH1000_TetR no antibiotic, Blue = SH1000_TetR TET, Green = SH1000_TetR DOX; normoxia = solid line, hypoxia =dashed line.

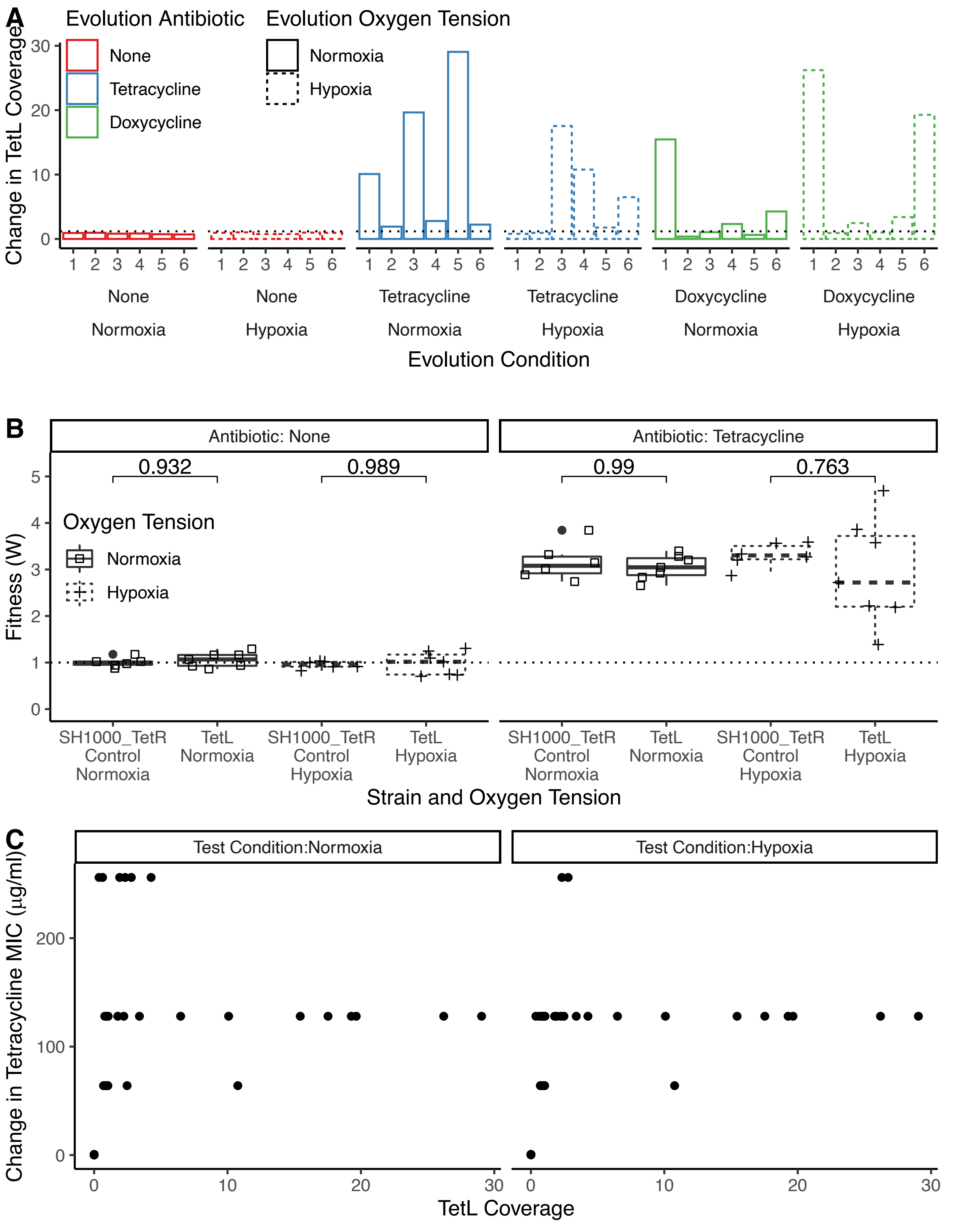

Supplementary Figure 5: Number of duplications, relative fitness and MIC of evolved clones carrying TetL duplications

A) Change in the TetL mean sequencing coverage compared to the mean coverage across the entire genome. 1-6 represent the 6 different SH1000_TetR clones in each

evolution condition. Dashed black line at 1.18 shows mean coverage of TetL in SH1000_TetR ancestor. Evolution conditions are: red=no antibiotic, blue=TET, green=DOX, solid line=normoxia, dashed line=hypoxia. B) Competition assay of kanamycin resistant SH1000 (SH1000_KanR) competed against clones with 26 times (n=4) or 15 times (n=3) TetL coverage compared to its ancestor (SH1000_TetR) in normoxia or hypoxia +/- TET (0.125μg/ml) for 24h starting from a 1:1 ratio performed in triplicate. Dashed line at 1 indicates no fitness cost, <1 show a fitness advantage over SH1000_KanR. Statistical analysis by 2-way ANOVA with Tukey's multiple comparisons. C) MICs measured in TET prepared by serial dilutions of antibiotics in equilibrated BHI broth in normoxia or hypoxia in 96 well plates. Bacteria were grown overnight before dilution to OD_{600}=0.05 before adding to TET dilutions. Following 20h incubation MIC cut-offs were defined as the lowest antibiotic concentration with no visible bacterial growth. n=6 clones evolved in each evolution condition. TetL Coverage calculated as in A.

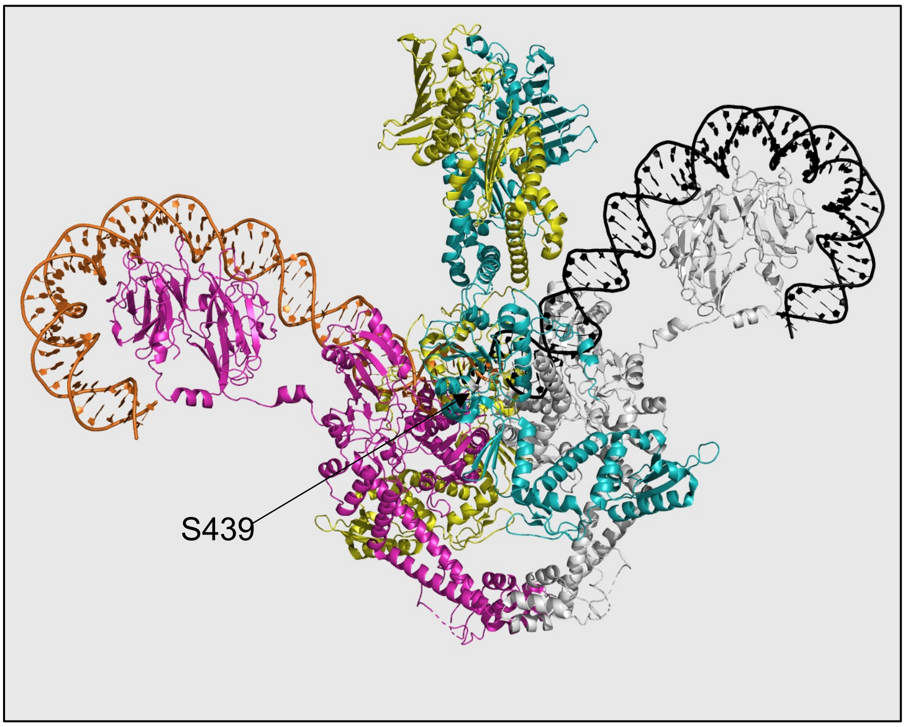

Supplementary Figure 6: DNA Gyrase with Ser439 mutation

Total structure of computational structural analysis of Ala439Ser DNA gyrase B mutation. Grey=chain A, cyan= chain B, pink= chain C, yellow= chain D, orange/black=DNA before/after cleavage.

Supplementary Table 3: Bacterial Strains used in this Study

Strain	Description	Reference
SH1000	*S. aureus* 8325-4 derived strain	(1)
SH1000_TetR (GMSA017)	SH1000 *lysA*::pGM070 (TetR) *lysA*+	(2)
SH1000_KanR (GMSA016)	SH1000 *lysA*::pGM072 (KanR) *lysA*+	(2)
SH1000_EryR (GMSA015)	SH1000 *lysA*::pGM068 (EryR) *lysA*+	(2)

References

1. M. J. Horsburgh *et al.*, sigmaB modulates virulence determinant expression and stress resistance: characterization of a functional rsbU strain derived from Staphylococcus aureus 8325-4. *Journal of bacteriology* **184**, 5457-5467 (2002).
2. G. McVicker *et al.*, "Clonal Expansion during Staphylococcus aureus Infection Dynamics Reveals the Effect of Antibiotic Intervention" in PLoS Pathog. (2014), vol. 10. 0).
